# A Maturity Matrix for Nurse Leaders to Facilitate and Benchmark Progress in Genomic Healthcare Policy, Infrastructure, Education, and Delivery

**DOI:** 10.1111/jnu.12586

**Published:** 2020-06-27

**Authors:** Emma Tonkin, Kathleen A. Calzone, Laurie Badzek, Caroline Benjamin, Anna Middleton, Christine Patch, Maggie Kirk

**Affiliations:** 1 Associate Professor of Genomics Healthcare, University of South Wales, Pontypridd, Rhondda Cynon Taff, Wales, UK; 2 *Xi*, Research Geneticist, National Institutes of Health, National Cancer Institute, Center for Cancer Research, Genetics Branch, Bethesda, MD USA; 3 *Alpha Rho*, Nu Omega, Dean and Professor, Penn State College of Nursing, Pennsylvania State University, University Park, PA USA; 4 Genetic Counsellor, Liverpool Women’s NHS Hospital Trust, Liverpool, England UK; 5 Head of Society and Ethics Research, Connecting Science, Wellcome Genome Campus; and Professor, Faculty of Education, University of Cambridge, Cambridge, Cambridgeshire, England UK; 6 Clinical Lead for Genetic Counselling, Genomics England, London, UK; Principal Staff Scientist, Society and Ethics Research, Connecting Science, Wellcome Genome Campus, Cambridge, Cambridgeshire; and Visiting Professor, Sheffield Hallam University, Sheffield, South Yorkshire UK; 7 Senior Author, Emeritus Professor of Genetics Education, University of South Wales, Rhondda Cynon Taff, Pontypridd, Wales, UK

**Keywords:** Genomics, maturity matrix, nursing, nursing leadership, strategy

## Abstract

**Purpose:**

Nurse leaders driving strategic integration of genomics across nursing need tools and resources to evaluate their environment, guide strategies to address deficits, and benchmark progress. We describe the development and pilot testing of a self-assessment maturity matrix (MM) that enables users to benchmark the current state of nursing genomic competency and integration for their country or nursing group; guides the development of a strategic course for improvement and implementation; and assesses change over time.

**Design:**

Mixed-methods participatory research and self-assessment.

**Methods:**

During a 3-day workshop involving nursing experts in health care and genomics, a genomic integration MM grid was built by consensus using iterative participatory methods. Data were analyzed using descriptive techniques. This work built on an online survey involving the same participants to identify the critical elements needed for “effective nursing which promotes health outcomes globally through genomics.”

**Findings:**

Experts from 19 countries across six continents and seven organizations participated in item development. The Assessment of Strategic Integration of Genomics across Nursing (ASIGN) MM incorporates 55 outcome-focused items serving as subscales for six critical success factors (CSFs): education and workforce; effective nursing practice; infrastructure and resources; collaboration and communication; public/patient involvement; policy and leadership. Users select their current circumstances for each item against a 5-point ordinal scale (precontemplation to leading). Nurses representing 17 countries undertook matrix pilot testing. Results demonstrate variation across CSFs, with many countries at the earliest stages of implementation.

**Conclusions:**

The MM has the potential to guide the strategic integration of genomics across nursing and enables additional assessments within and between countries to be made.

**Clinical Relevance:**

Nurse leadership and direction are essential to accelerate integration of genomics across nursing practice and education. The MM helps nurse leaders to benchmark progress and guide strategic planning to build global genomic nursing capacity.

Genomics is here to stay, bringing with it a new paradigm of health care through the emergence of precision medicine (Ginsburg & Phillips, 2018). The Global Genomics Nursing Alliance (G2NA, www.g2na. org) recognizes the vital role that nurses can and do play in delivering genomic health care and the centrality of nursing leadership in enabling the integration of genomics into nursing care. The G2NA landscape analysis highlights wide variation in nurse preparation and involvement in genomics globally, with lack of visible nursing leadership being a barrier to progress (Calzone et al., 2018a). Limited availability of supporting resources such as competency or curriculum guidelines, educational tools, and specialist genomics nursing societies (Calzone et al., 2018b) compounds the challenge for nurse leaders who want to take a strategic approach to integrating genomics into nursing practice in their sphere of influence. To accelerate genomic integration in nursing education and practice, the G2NA sought to produce a flexible and accessible tool (maturity matrix [MM]) to guide global nurse leaders and facilitate benchmarking of the current state of nursing genomic competency and integration, and measure change over time. This article describes the development and pilot testing of the MM.

## Background

### Emergence of Precision Health Care

Ginsburg and Phillips (2018) outlined how, with costs of DNA sequencing declining, genomics and genome-based technologies are being used increasingly in diagnostic and predictive testing. New genomics approaches are forming the basis for reframing disease taxonomy, enabling more precise screening and earlier disease detection, with therapeutic options guided by individual genomic variations. With these advances, healthcare strategies are shifting from acute intervention to genotype-guided risk management (including lifestyle choices), tailored health monitoring, and disease management. This facilitates therapeutic decisions to be targeted more precisely and is the basis of precision health care. Bilkey et al. (2019) presented examples of 12 applications of genetic testing from pre-conception to post-mortem molecular genetic testing, in clinical and wider healthcare settings. In addition, Bilkey et al. highlighted the challenges around the use of personal genomic data, including data sharing, informed consent, and dealing with unexpected findings. Clinically available genomic applications have immediate implications for nurses. These include having the capacity to provide patient/family education or informed consent; the need to understand clinical testing processes and possible test outcomes; to be aware of service pathways; and to appreciate ethical, legal, and socio-economic aspects.

### Genomics Implementation and Collaboration

Translational genomics activities span initial basic science discoveries right through to population health impact studies (Schully & Khoury, 2014). They include development and evaluation of candidate applications and assessment of projects to implement and integrate genomics into routine clinical practice. Ginsburg and Phillips (2018) noted that translation of applications into clinical practice lags behind the pace of discovery. However, where patients’ genomic information is being used in clinical care, Manolio et al. (2013) found that such institutions tended to work in isolation rather than collaboratively. In their appraisal, Stark et al. (2019) summarized the increasing use of genomics in research and clinical practice and described the approaches and progress being made towards integration in four (high-income) countries. Whilst the approaches in those countries may not be relevant to low- or middle-income countries, the benefits of sharing experiences and resources may increase the efficiency and effectiveness of implementation. Information on wider translation across global healthcare systems remains scarce.

Limited evidence exists for genomic implementation into practice and education considering varied conditions and contexts (Calzone, Jenkins, Culp, & Badzek, 2018; Jenkins & Calzone, 2014). Taylor et al. (2019) outlined a protocol for a study aimed at integrating genomics into clinical practice in 29 health systems in one country. The protocol aims to understand the complex adaptive systems needed for genomic practice integration, including clinical care effectiveness; policy and service provision; and individual level behavior change. Findings from this study will produce an evidenced-based toolkit to facilitate translating genomics into health care. It will expand existing strategies and resources for other health professionals and add to the nursing-specific clinical implementation toolkit from the Method for Introducing a New Competency: Genomics (MINC) study (Jenkins et al., 2015 https://genomicsintegration.net/). Given the scope and rapid expansion of evidence-based genomic clinical applications, these toolkits provide evidence-based strategies and resources for utilization in other countries to facilitate genomic translation to improve health outcomes. As such, the global nursing collaborative G2NA is helping to compile and disseminate tools, facilitate collaborative research, and contribute to best practices to accelerate the implementation of genomics into nursing practice.

### Nursing and Genomics

Specialist genetics services, with their focus on the rarer inherited and chromosomal conditions, have long been the province of specially trained genetic nurses and counselors. However, all nurses care for individuals and families affected by inherited conditions as they encounter them in everyday practice. Precision medicine further broadens the focus across all health care as advances in understanding the genomic component of common diseases, targeted surveillance, and treatment selection have implications for all specialties and healthcare providers. Nurses, as the largest global health professional workforce, who spend the most time with patients in both acute and community settings, and deliver direct care as well as patient family education, are essential for genomic translation into clinical practice. There have been significant and sustained efforts internationally to articulate roles and genomic competencies that nurses in any specialty and level of academic preparation should be able to demonstrate when implementing genomics into nursing practice. Genomic nursing competency contributes to achieving the potential of genomic science and technologies, to meet the needs of patients or families, and to improve healthcare quality, efficacy, and safety. However, evidence of variability in capacity, capability, and confidence in genomics has been well documented even in countries with sustained genomic nursing competency efforts (Calzone et al., 2018a; Jenkins, 2019).

Kirk, Calzone, Arimori, and Tonkin (2011) explored perspectives on nursing and integration of genomics across 10 countries. They found that nursing leadership and engagement of senior nurses within government and nursing regulation was fundamental for nurses to play a part in delivering genomic health care. However, nurse leaders faced resource constraints to support change initiatives (Jenkins, 2019). Nursing leaders having access to established resources will reduce the duplication of effort, facilitate learning from each other, and enable collaborations for evidence generation of best practices. In turn, this will help accelerate genomic nursing integration.

### The Maturity Matrix

An MM is used to assess the progress of development of an organization or unit over time, identify development needs, and stimulate quality improvement efforts (Buch, Edwards, & Eriksson, 2009). The MM approach was originally developed within health service settings by Elwyn et al. (2004) to assess practice developments in primary care settings and to promote communication and learning. They found it provided insight into improvement needs and prioritization, with high face validity. Subsequent studies adapted MMs for use in several European countries, such as the International Family Practice Maturity Matrix (Elwyn et al., 2010). A version developed for dentistry (the Maturity Matrix Dentistry) includes 12 domains (Barnes et al., 2012). In the United Kingdom, Cardiac Genetics Nurses used an MM to track progress in the development of inherited cardiac conditions services. Its five domains provided a comprehensive assessment framework (Kirk et al., 2014).

An MM consists of a series of concepts or domains, displayed as a grid that incorporates the sub-components (termed key enablers) of each domain. Each row shows a criterion, or indicator, of a key enabler, with the expected development stage of that indicator shown for a given stage of maturity (Table S1). The maturity stages are incremental and assume that the outcomes of the previous stages have been met. While maturity matrices have a common structure and are based on self-assessment, the content is flexible and adaptable and can be developed specifically for the needs of a particular service, group, or organization (Maier, Moultrie, & Clarkson, 2012). MMs can operate at micro (e.g., local teams), meso (e.g., healthcare organization), and macro (e.g., national/international) levels. At each level, the MM provides a framework for assessment and advancement of capability and capacity. Importantly, the MM also provides a common measurement system for healthcare systems delivering care regardless of country-specific factors.

### Aims

Nurses worldwide lack confidence and competence in teaching genomics and using genomics in practice (Anderson et al., 2015). Resources are limited, and progress in genomic implementation in education and practice varies widely (Calzone et al., 2018a, 2018b). To address these deficits, strategic approaches are needed to guide change initiatives in genomics and measure outcomes. Our primary aim was to develop and pilot test a new MM for nursing that assesses the status of genomics integration within a nursing group (e.g., country, hospital, academic program).

## Methods

Creating the MM was predicated on three assumptions. The framework needs to (a) be broad and flexible to accommodate different socio-economic, political, and cultural factors, and the dynamic nature of both genomics and nursing; (b) consider the broader nursing context; and (c) have evaluation outcomes that are wide ranging to accommodate different stages of maturity. The Faculty of Life Sciences and Education Ethics Committee, University of South Wales, reviewed and approved this project (Ref 2017ETMK1201).

### Participants

A purposive sample of experts with senior nursing leadership roles and expertise in health care, nursing, education, policy, and genomics, and a representative of a national genetic conditions advocacy organization attended a 3-day workshop to discuss the formation of G2NA. Expertise in genomics was not essential. For the purpose of the event, individuals primarily represented either their country or an organization. All participants had the opportunity to contribute to Phases 1 and 2 of MM development. Country representatives pilot tested the MM. Countries with more than one person in attendance submitted a single response.

### Procedures

MM development took place over three broad phases (Figure S1).

#### Phase 1: Selection and refinement of critical success factors (CSFs).

To identify potential key themes for constructing the MM, we invited G2NA workshop participants through Online Surveys (www.onlinesurveys.ac.uk, formerly BOS) to propose a maximum of six responses to the following question: “What are the core essential elements for effective nursing which promotes health outcomes globally through genomics?” Items were coded independently by two authors and categorized through discussion. Consensus on the final themes (CSFs) was reached with participants through discussion and real-time electronic voting at the workshop.

#### Phase 2: Development of MM key enablers and incremental scales.

At the workshop we used an iterative, consensus-building approach (Kirk et al., 2014) that drew on Liberating Structures methods (Lipmanowicz & McCandless 2014) to establish the key enablers and incremental scales. Participants worked in mixed small groups on one CSF at a time to identify the underpinning key enablers and the indicators that could be used to illustrate the achievement of each key enabler. Participants formulated what progress against each indicator might be expected over time and considered what measures could be used as evidence of progress. All information was captured on worksheets. Iteratively, groups rotated through each CSF, reviewing prior group comments. One workshop organizer acted as a facilitator for each CSF remaining with the worksheet, providing clarification and context to comments of prior groups. In the final round, the original group was able to review all comments and revisions made by subsequent groups to its initial outline.

#### Phase 3: Refinement and Feasibility Pilot.

The key enablers, indicators, progress descriptions, and measures generated by participants were reviewed, synthesized, and refined post-workshop by the organizers to create the MM now entitled Assessment of Strategic Integration of Genomics across Nursing (ASIGN). Participants were then invited to pilot test ASIGN online by self-assessing for each indicator the current situation of their country, to the best of their knowledge and in collaboration with others as appropriate. Two paper-based versions of ASIGN were also created. The ASIGN Self-Assessment Document is the same as the online version and contained blank cells for users to mark the assessed stage of change. Free text boxes captured comments about measures used, with supporting evidence and further general comments on the likely pace of progress for each CSF. The ASIGN Reference Document (Figure S2) is for information only, providing explanation about completion, with example measures that could be used for each key enabler and definitions of the five stages of maturity used (Table 1). Timescales were not defined for movement through stages 1 to 5, since these are dependent on wider local and national contexts. We also acknowledged that there is not necessarily an equal distance between the stages of maturity. Feedback was requested on whether ASIGN represented the discussion and content generated at the workshop as well as participants’ experience completing the country assessment, including whether the maturity progression followed a logical path, how confident they felt in their assessments, and the time taken to complete ASIGN.

### Analysis

Data were exported into Excel and frequencies calculated for the number of countries at each stage of maturity for individual key enablers. The same approach was used to calculate frequencies on the participant feedback on experience of utilizing and completing ASIGN. Stages of maturity were coded 0 to 4 (0 = *pre-contemplation* and 4 = *leading*). The total scores for each CSF for individual countries were calculated, as were the percentages of the maximum scores for each CSF. Data for all countries were then combined to calculate an overall distribution for each CSF. Cluster analysis to group the CSFs according to similarity in assessment was performed using the complete linkage method (Euclidean distance), utilizing each country’s mean score.

## Results

### Participants and ASIGN Development

A total of 30 individuals (including the 6 workshop organizers) with a range of professional backgrounds, most with some genomic expertise, representing 19 countries across six continents and seven organizations were involved in the development or pilot testing of ASIGN (Table 2). Countries and organizations were each represented by one person, with the exception of the United Kingdom and the United States (workshop organizers), and Health Education England. Fifteen Phase 1 survey respondents generated 84 core elements of genomic health care, which was reduced to 63 after removal of duplicates. Thematic analysis resulted in seven categories. Participant discussion and voting (not including the organizers) resulted in six distinct CSFs (A–F) necessary for delivering effective nursing care that promotes improved health outcomes through genomics (Table 3). Phase 2 group work and post-workshop refinement produced a total of 19 key enablers and 55 indicators across the six CSFs (see Table 3). Full details of the CSFs, key enablers, and indicators that make up ASIGN are provided in Table S2.

### ASIGN Pilot Testing

Of 19 eligible country representatives, 17 completed the pilot. One country representative forwarded the pilot to a graduate student who also completed the assessment; the student responses were excluded from the analysis. The U.S. pilot was completed jointly by both representatives. Only one U.K. participant completed that country’s assessment.

**Figure 1.**
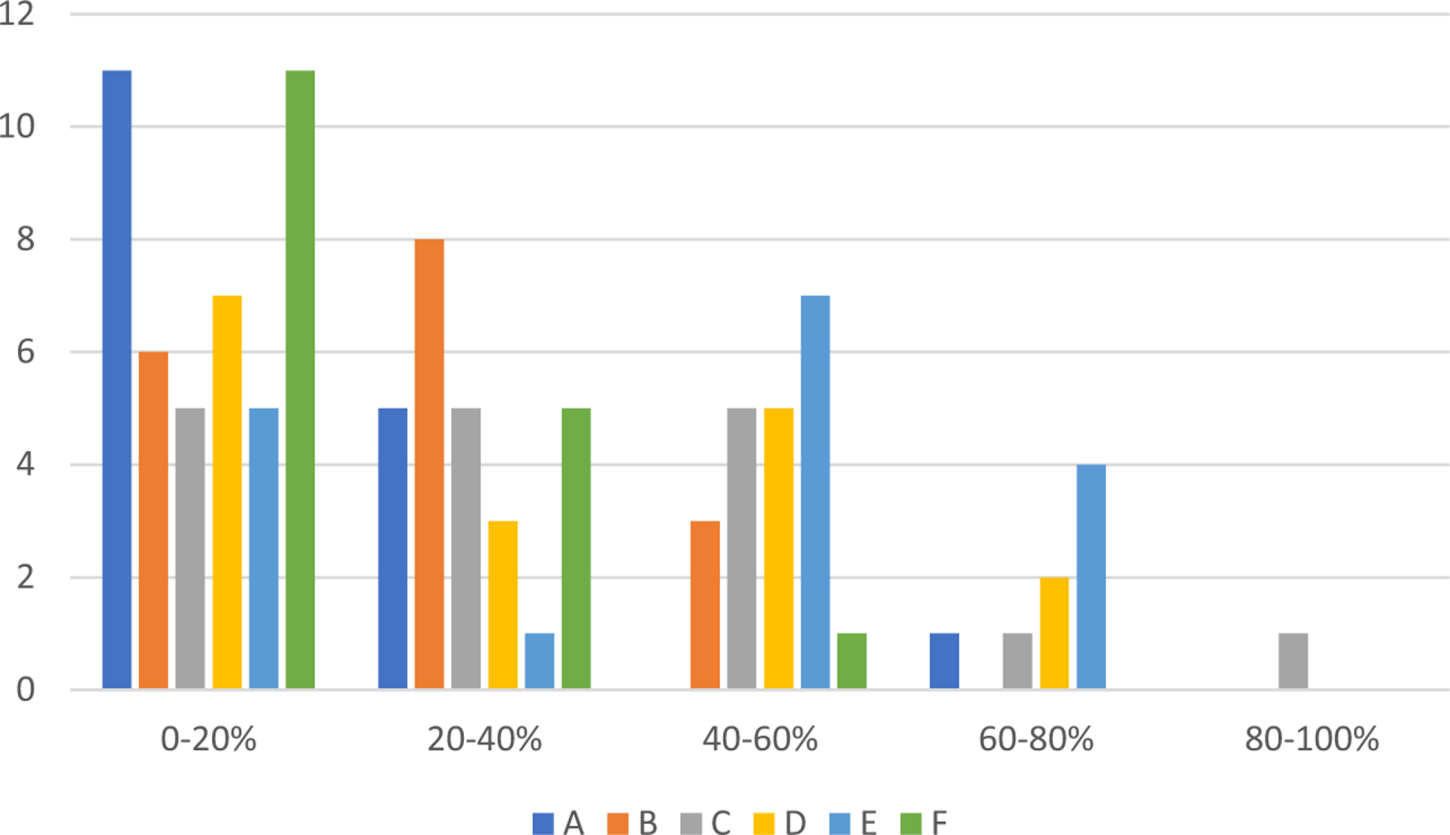
Number of countries and percentage of the total maximum score achieved for each critical success factor in Assessment of Strategic Integration of Genomics across Nursing (ASIGN). A = enhanced education and workforce development; B = effective nursing practice; C = infrastructure and resources that support incorporation of genomics in practice; D = interprofessional collaboration and communication; E = family- and community-focused care; F = health care transformed through policy and leadership.

**Figure 2.**
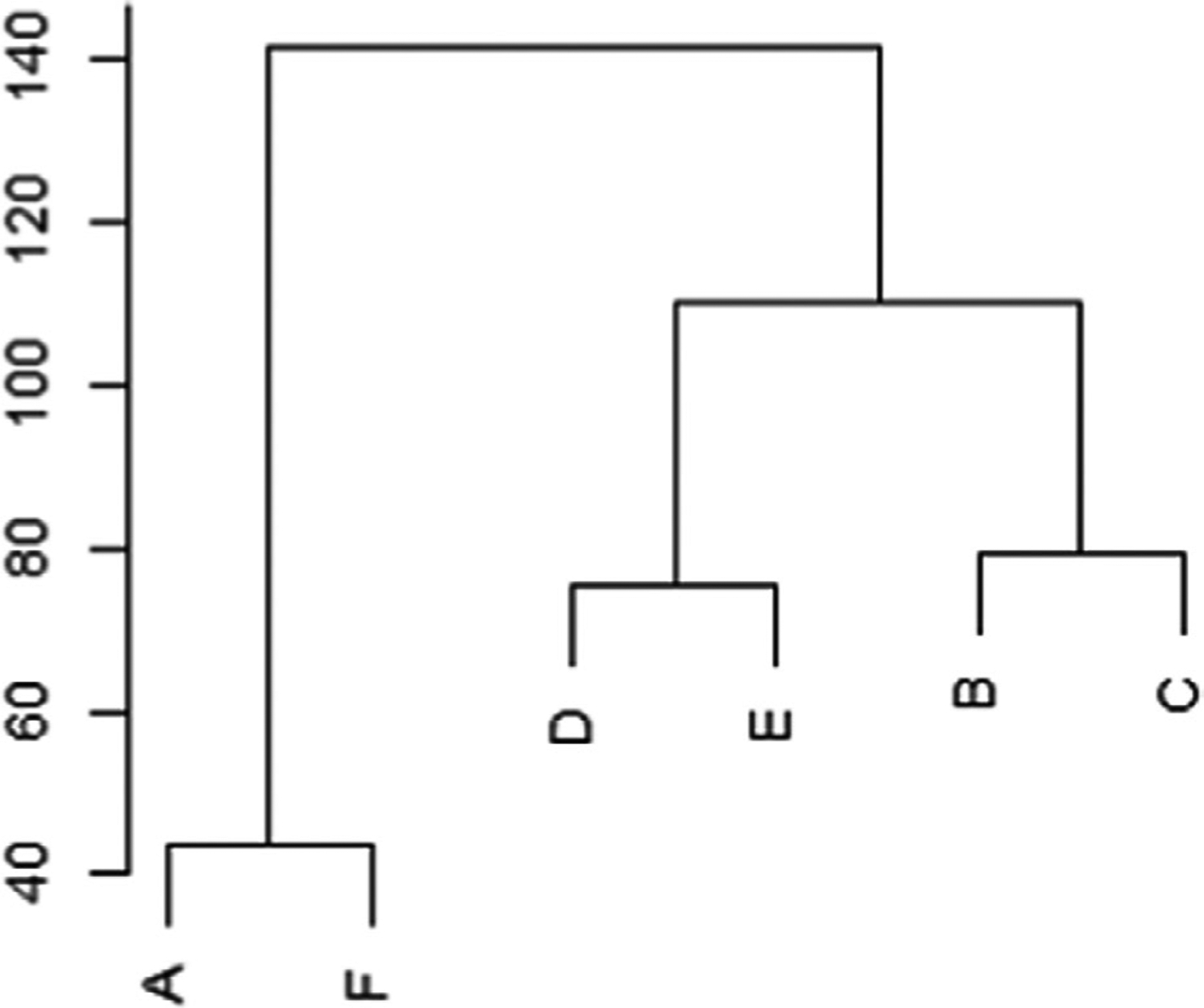
Cluster analysis of the critical success factors (Euclidean distance). A = enhanced education and workforce development; B = effective nursing practice; C = infrastructure and resources that support incorporation of genomics in practice; D = interprofessional collaboration and communication; E = family- and community-focused care; F = health care transformed through policy and leadership.

Country assessments (percentage of maximum score) for each CSF (Figure 1) illustrate the variability and distribution within and between CSFs. Cluster analysis (Figure 2) shows similarity in responses from countries to CSFs A and F, for which respondents located most indicators at the two earliest stages of maturity (“pre-contemplation” and “awareness and planning”). Figure 2 also illustrates the dissimilarity of responses to A and F compared to the other four CSFs, where indicators have a greater spread across all stages of maturity.

The number of countries at each stage of maturity for the 19 key enablers is illustrated in Figure S3. In summary, for CSF A (enhanced education and workforce development), most countries self-assessed as being at stage 1 (precontemplation) or 2 (awareness and planning). Stage 5 (leading) was selected by only one respondent, for two of the four indictors within the key enabler “culture of positive attitude towards nursing and genomics.” CSF B (effective nursing practice) had a greater spread across the maturity stages for the key enablers “evidence-informed practice” and “ethical and safe practice,” with the indicator “policies regarding the confidentiality and use of genomic information” (B4–2; see Table S2) at stages 4 (embedded) or 5 in nine countries. However, only two countries assessed both indicators for “clearly defined patient outcomes” as being at stage 3 (active commitment) or greater. The picture for CSF C (infrastructure and resources that support incorporation of genomics in practice) is more variable. A range of maturity for the indicators of “service capacity” was observed, with five countries assessing all four indicators at stages 4 or 5. In contrast, most countries reported being at stage 1 for the three indicators of “human resources that support nursing career potential.” Within CSF D (inter-professional collaboration and communication), countries appear to be at a range of maturity for “strong working relationships” and “effective communication,” with evidence of practice that is embedded or leading. However, the indicators for “collaboration across boundaries to share genomics knowledge, expertise, and resources” are still at stages 1 or 2 for most countries. Responses to CSF E (family and community focused care) indicate some movement towards more mature stages of practice, with some evidence of at least embedded activity for all indicators. CSF F (healthcare transformed through policy and leadership) is composed of nine indicators, with most assessments at stages 1 or 2. For five nursing leadership indicators (F1.3, F2.1, F2.2, F3.1, and F3.3; see Table S2 for details) there was no progress in any country beyond active commitment (stage 3).

All respondents indicated that the matrix followed a logical progression across each indicator. All of those who attended the workshop responded that ASIGN represented the discussions held. Some variation in self-confidence when completing the assessment was noted, with 11 of 17 (65%) being either very confident or confident of accuracy and consistency across their country. However, three people raised concerns about potential regional differences, with one commenting, “Since the wealth gap between urban and rural areas is very wide, it will be very challenging for the MM to benchmark progress in such a heterogeneous [country] serving different populations.” Most did not or could not provide estimates of the timescales in which they anticipated progress for each of the CSFs, although one respondent predicted at least a 5- to 10-year timescale in their country before significant progress could be made in any of the CSFs.

Comments about completing the assessment were generally positive, but for some the exercise was more time consuming, depending on level of wider context, access to corroborating evidence, and first language. Whilst some had found the supporting documents useful, one commented that it “was a bit long and cumbersome.”

## Discussion

ASIGN provides a systematic approach to managing the strategic integration of genomics across nursing and is the first MM developed for this purpose. It fosters comprehensive evaluation from the outset, informs revision of current strategies or development of new ones, and provides evidence to support targeting of resources. Ongoing self-assessment and evaluation will help make the nursing role more visible and promote a greater appreciation of the nursing contribution to genomic healthcare.

The essential elements that need to be in place for nurses to be able to deliver effective care that integrates genomics into standard practice are explicit within ASIGN. The tool provides the basic framework to guide further development in genomics, including guiding academic and continuing education. By using the matrix as a framework for assessment, groups, organizations, or countries can use ASIGN to benchmark themselves at a starting point to inform a plan for progress. The tool is nimble, as users can be a country or region within a country, or an organization such as a hospital, professional body, or government. Focusing on outcomes, users of ASIGN can capture both the current status and change over time. Comparisons between indicators and stage of maturity (i.e., high vs. low maturity) can help users identify areas where work needs to be focused. Individuals can identify the most appropriate measures (evidence) for each indicator, thus offering some flexibility, in recognition that there will be wide variations in resources, infrastructure, and service provision across countries, regions, or organizations. Acknowledging the variations between countries (Calzone, Jenkins, et al., 2018) influenced by political, fiscal, and environmental aspects beyond their control, data on individual countries are not presented and comparisons between countries have not been made.

This pilot study data provide insight into the current status and role of nurses in relation to genomics. One respondent commented that it would be 5 to 10 years before genomics becomes a nursing priority in their (European) country. The scale and nature of change needed to promote and achieve genomics integration is substantial, and ASIGN reflects this in requiring appraisal across six domains, with 19 key enablers and a total of 55 indicators. To facilitate change, nurse leaders need to consider not just the content of ASIGN, but also the wider context for change and the change process itself. For this reason, ASIGN has been set into a wider facilitative framework (roadmap) by G2NA to provide practical guidance (Tonkin et al., 2020).

## Limitations

ASIGN incorporates multiple concept domains, key enablers, and indicators that are not mutually exclusive, with inherent overlap. To make the MM feasible for completion, similar related items associated with the same indicator were grouped together. For example, C1.4 (bioinformatics and IT support for variation interpretation, data storage, retrieval, and reporting are in place; see Table S2) grouped four closely related items: interpretation, storage, retrieval, and reporting. The intent for this indicator and others like it is to have an over-arching assessment on the state of maturity, which considers each item to render an overall determination for the indicator. We recognize that a group performing an assessment may identify different stages of maturity for individual items, and this could cause confusion on how to establish the overall stage of maturity. However, we had to balance the need to retain enough detail (to guide development in an area) with achieving an MM that was realistic in size and scope for usability. Pilot testing did not reveal this to be a significant problem, with just one country identifying the issue. As greater maturity is achieved for an item, this issue could be a greater concern for users.

The next steps are to conduct additional testing for further refinement. At the Nursing, Genomics, and Healthcare international conference taking place in July 2021, completion of ASIGN by a larger group of participants is planned. These data will be used to perform factor analysis, which is expected to help in coalescing or removing items thereby further refining ASIGN. Additionally, a subset of volunteers will be recruited to participate in a talk aloud session with facilitators that will audio record responses to targeted usability and language clarity questions. Both sets of participants will be asked to complete ASIGN a second time in a short interval following the conference to do preliminary test-retest reliability assessment. These assessments will inform revisions.

## Conclusions

ASIGN is the first MM designed and piloted to assess the integration of genomics into nursing. ASIGN is based on expected outcomes, predefined by key stake-holders that consisted of global nursing leaders from practice, education, and research, with and without genomic expertise. The matrix is outcome focused, capturing both stage of maturity and continuity, and enables assessment of changes over time. ASIGN is amenable for use in a variety of environments, including clinical, educational (nursing school), and professional (nursing organization), and at different scales, from hospital department to countrywide assessments. ASIGN helps answer the question “What does effective nursing which promotes health outcomes globally through genomics look like?” Coupled with the companion roadmap (Tonkin et al., 2020), ASIGN can be used as a reference point to inform further strategic genomic implementation development and measure change over time.

## Supplementary Material

Supplemental tables

## Figures and Tables

**Table 1.** Stages of Maturity and Corresponding Definitions

Stage of maturity	Definition
Pre-contemplation	There is little or no evidence of the indicator from any appropriate, identified measure that has been selected by the assessor.
Awareness and planning	There is some evidence of awareness of the need to address the indicator and planning is underway to do so, although there may be little or no evidence of the indicator being in place from any appropriate identified measure.
Active commitment	Plans are being implemented and there is some evidence that progress is being made.
Embeddec	Substantial progress has been made and the indicator is demonstrably established across most practice fields.
Leading	The indicator is embedded as the norm and the unit being assessed champions good practice and leadership in engaging others in demonstrating the indicator and promoting continuous improvement.

**Table 2.** Countries or Organizations Participating in ASIGN Development or Pilot Testing

Country or organization	Number of participarts	Educator (E), clinician (C), organization leader (OL)	Genomic expertise Yes/No	Phase 1 CSF survey Yes/No	ASIGN development participatior Yes/No	Pilot test participatior Yes/No
Australia	1	E	Yes	No	No	Yes
Brazil	1	E, C	Yes	Yes	Yes	Yes
Canada	1	E	Yes	Yes	Yes	Yes
Columbia	1	E	No	Yes	Yes	Yes
Germany	1	E	No	Yes	Yes	Yes
Hong Kong	1	E	Yes	Yes	Yes	Yes
Israel	1	E	Yes	No	Yes	Yes
Japan	1	E	Yes	Yes	Yes	Yes
Mexico	1	E	Yes	No	Yes	Yes
Netherlands	1	C	Yes	No	Yes	Yes
Norway	1	C	Yes	No	Yes	No
Nigeria	1	E	No	No	No	Yes
Pakistar	1	C	Yes	Yes	Yes	Yes
South Africa	1	E	No	No	Yes	No
Switzerland	1	E	Yes	Yes	Yes	Yes
Taiwan	1	C	Yes	Yes	Yes	Yes
Turkey	1	E	Yes	Yes	Yes	Yes
United Kingdom	5	E (n = 2) and C (n = 3)	Yes	Yes *(n* = 2)	Yes	Yes
United States	2	E, C	Yes	Yes *(n* = 1)	Yes	Yes
EBMG	1^a^	OL, C	Yes	No	Yes	No
GAUK	1	OL	Yes	No	Yes	No
HEE	2	OL	Yes	No	Yes	No
ICN	1	OL	Yes	No	Yes	No
ISONG	1	OL, C	Yes	Yes	Yes	No
STTI	1	OL, E	Yes	Yes	Yes	No
NHGRI	1^b^	C	Yes	No	Yes	No

*Note*. ASIGN = Assessment of Strategic Integration of Genomics across Nursing; EBMG = European Board of Medical Genetics; GAUK = Genetic Alliance UK; HEE = Health Education England; ICN = International Council for Nurses; ISONG = International Society of Nurses in Genetics; NHGRI = National Human Genome Research Institute (U.S.A.); STTI = Sigma Theta Tau International.

a Also represented the United Kingdom.

b Also represented the United States.

**Table 3.** The Number of Key Enablers and Indicators Across the Six Critical Success Factors (A–F) of ASIGN

Critical success factor	Key enablers	Indicators
A. Enhanced education and workforce	3	9
development		
B. Effective nursing practice	4	12
C. Infrastructure and resources that	3	11
support incorporation of genomics in		
practice		
D. Interprofessional collaboration ano	3	7
communication		
E. Family- and community-focused care	3	7
F. Health care transformed through policy	3	9
and leadership		
Total	19	55

*Note*. ASIGN = Assessment of Strategic Integration of Genomics across Nursing.

## Clinical Resources

- Genetics/Genomics Competency Center. https://genomicseducation.net/
- Global Genomics Nursing Alliance. https://g2na.org/
- International Society of Nurses in Genetics. https://www.isong.org/
