# Supplementary material for: A Maturity Matrix for Nurse Leaders to Facilitate and Benchmark Progress in Genomic Healthcare Policy, Infrastructure, Education, and Delivery
Source: J Nurs Scholarsh. Author manuscript; Available in PMC 2020 Dec 8. (PMC7721977; doi:10.1111/jnu.12586)
Supplement: Supplemental tables [file NIHMS1612233-supplement-Supplemental_tables.docx]

**Table S1. The Maturity Matrix: General Structure and Terms**

| **Critical Success Factor A** | | | | | | |
| --- | --- | --- | --- | --- | --- | --- |
| **Key enabler**s | **Indicators** | **Benchmarks**  Increasing stage of maturity >>>>>> | | | | |
|  |  | **Stage 1** | **Stage 2** | **Stage 3** | **Stage 4** | **Stage 5** |
| Key enabler 1 | Indicator 1 | Little or nothing of the measure is in place | Awareness of need; planning underway although little or no measure may be evident | Plans are being implemented; progress is being made; some of the measure is in place | Substantial progress is being made. Most of the measure is in place. | All of the measure is in place; leadership is evident in taking the measure forward. |
|  | Indicator 2 |  |  |  |  |  |
| Key enabler 2 | Indicator 3 etc. |  |  |  |  |  |
| Measures used as evidence of stage attained | | e.g. Nurse registration regulations; nursing competency standards, care pathways, policy documents | | | | |

**Definition of terms:**

Critical Success Factor: A concept/domain that identifies an essential characteristic of a mature group/organisation in the area of practice that is being assessed (e.g. effective genomic nursing).

Key Enabler: Individual sub-component of a Critical Success Factor.

Indicator: Item that can be measured and used to inform location on the grid for a specific Key enabler.

Measures: provide the evidence for location of benchmark stage selected.

**Figure S1. ASIGN Maturity Matrix Development Process**

Key. ^1^ Items generated in response to the question ‘What are the core essential elements for effective nursing which promotes health outcomes globally through genomics?’

^2^ activities via iterative small group work, interspersed with whole group review: ‘Shift and Share’ (Lipmanowicz, 2014)

^3^ activities using a modified ‘Panarchy’ approach, working individually, then in small groups prior to whole group review (Lipmanowicz, 2014)

Phase 1: Selection and refinement of CSFs

Phase 2: Development of MM key-enablers and incremental scales

Phase 3: Refinement and feasibility pilot


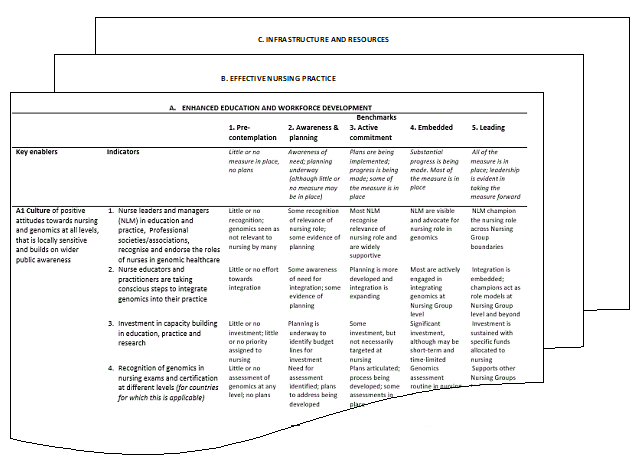


**Figure S2. ASIGN sample page to illustrate layout and stages of maturity.**

**Table S1: Critical Success Factors (A-F) with key enablers and indicators of Assessment of Strategic Integration of Genomics Across Nursing (ASIGN) Maturity Matrix**

| **Key enablers** | **Indicators** |
| --- | --- |
| **A. Enhanced education and workforce development** | |
| **A1** **Culture** of positive attitudes towards nursing and genomics at all levels, that is locally sensitive and builds on wider public awareness | 1. Nurse leaders and managers in education and practice, Professional societies/associations, recognise and endorse the roles of nurses in genomic healthcare |
|  | 1. Nurse educators and practitioners are taking conscious steps to integrate genomics into their practice |
|  | 1. Investment in capacity building in education, practice and research |
|  | 1. Recognition of genomics in nursing assessments and certification exams at different levels *(for countries for which this is applicable)* |
| **A2 Core competencies** in genomics which are culturally sensitive, responsive and engender critical analytic skills; realistic at the macro level taking into account the existing nursing workforce, across all levels, roles, and clinical specialties | 1. Genomic competencies agreed and embedded into curricula for pre-qualifying/student nurse courses |
|  | 1. Genomic competencies integrated into continuing professional development CPD courses at all levels and across specialties |
|  | 1. Competency framework is harmonised so standards are consistent across Units |
| **A3 Trained faculty**, including adjunct staff and clinical preceptors, who are confident, competent and collaborative in genomics | 1. Active support and encouragement for faculty to develop competence in genomics through accessing courses and other CPD opportunities |
|  | 1. Training is facilitated through accessible and relevant resources, developed and shared at institutional/national/international levels, endorsed by professional organisations |
| **B. EFFECTIVE NURSING PRACTICE** that is person/family centred, evidence-informed, safe, ethical and clearly defined | |
| **B1 Evidence informed practice** to enhance quality care | 1. Evidence based resources, including clinical support tools, are accessible and utilised |
|  | 1. Nurses leading and contributing to generation of evidence |
|  | 1. Evidence based service provision (commissioning) |
|  | 1. Existence of evidence based multidisciplinary care pathways for health conditions in which genomics is relevant. |
| **B2** **Clearly defined role** of the practising nurse in applying genomics in care delivery | 1. Health service employers specify minimum standards of core knowledge and skills sets in genomics for incorporation across the nursing workforce |
|  | 1. National recognition of nursing advanced practice (AP) role which incorporates genomics within the multidisciplinary team |
|  | 1. National recognition of the role of nurses within the specialty of clinical genetics services |
| **B3 Clearly defined patient outcomes** related to genomic care | 1. Health outcomes are agreed in partnership with patients and the multidisciplinary team and are established and evaluated for health improvement |
|  | 1. Patient reported outcome measures (PROMs) for genomic nursing are established and used |
| **B4 Ethical and safe practice** utilising genomics | 1. Ethical codes of conduct are demonstrably cognisant of genomics implications |
|  | 1. Policies are in place across healthcare settings regarding confidentiality and use of genomic information |
|  | 1. Continuing professional development in genomics is actively supported |
| **C.** **SUSTAINABLE INFRASTRUCTURE AND RESOURCES** that support incorporation of genomics into education and practice | |
| **C1 Service Capacity**  Genomics clinical and testing services are delivered to agreed and accredited standards, underpinned by sustainable infrastructure that includes human resources | 1. Genomic tests and services are available and accessible to all (equitable) |
|  | 1. Standards for clinical genomics services are comprehensive and evaluated (including clinic performance, clinical personnel and clinic provision/space) |
|  | 1. Standards for genomics laboratory services are comprehensive and evaluated (including testing methods, laboratory personnel and laboratory equipment and space) |
|  | 1. Bioinformatics and IT support for variation interpretation, data storage, retrieval and reporting are in place |
| **C2** **Political and financial investment** to support adequate and appropriate workforce development and sustainable genomics service delivery, with commitment at strategic policy levels | 1. Organsational/regional/national support for investment and development of genomic healthcare is evident at strategic planning level |
|  | 1. Allocated base funding (line item) in the budget for genomics education/ healthcare |
|  | 1. Budget addresses time and staffing for service delivery |
|  | 1. Budget addresses need for resources to support education (patient/family and healthcare provider) and/or clinical support tools. |
| **C3 Human Resources** that support nursing career potential: within the genomics sub-specialty; and for all nurses who incorporate genomics | 1. Unit plan for human resources required for genomic service delivery |
|  | 1. Allocated positions for nurses in genomics in the health care system |
|  | 1. Infrastructure is in place for nurses to research genomics and research its application to healthcare and nursing |
| **D. COLLABORATION AND COMMUNICATION** to create social capital to accelerate change | |
| **D1** **Strong working relationships** which are collaborative, may be multidisciplinary, and which result in positive outcomes, influence, impact and advocacy | 1. Examples of cooperative working relationships within and between organisations and countries are evident |
|  | 1. Services delivered reflect the multidisciplinary teams’ shared vision for genomic healthcare |
| **D2** **Collaboration across boundaries** to share genomics knowledge, expertise and resources to facilitate education and practice | 1. Nurses are actively seeking opportunities to collaborate |
|  | 1. Mechanisms are in place to facilitate interaction and information-sharing |
|  | 1. Social capital generated through networking is harnessed to create an environment for change |
| **D3 Effective communication** across stakeholder groups | 1. A common language in genomics is developed to facilitate dialogue, tailored appropriately to stakeholder group |
|  | 1. Communication is culturally sensitive, and respectful of mother tongue and time-zone differences |
| **E.** **PUBLIC AND PATIENT INVOLVEMENT** that is core to policy and practice | |
| **E1 Commitment to** enhance health literacy in genomics for the public, society and healthcare services through engagement and dialogue | 1. Awareness-raising activities through public and patient involvement (PPI) are encouraged and expected |
|  | 1. Opportunities for dialogues to stimulate increasing genomic engagement |
| **E2** **Access to services** | 1. Services are accessible, taking into account geography, awareness amongst ethnic groups, referral practices and socio-economic status |
|  | 1. Information about genomic services and pathways is easy to understand (for the patient, family) |
| **E3 Patient involvement and empowerment** (and family as part of the health care system) | 1. Effective partnerships nurtured between professionals and patients/families |
|  | 1. Relationships with other relevant groups (e.g. patient advocacy) exist and are valued |
|  | 1. Patients and families have appropriate knowledge and understanding of genomics to manage their own situation |
| **F. HEALTHCARE TRANSFORMED THROUGH POLICY AND LEADERSHIP** | |
| **F1 Shared vision** of the essential contribution of nurses to improved health outcomes through delivering genomic healthcare | 1. Vision for nursing and genomics articulated for broad audience (at professional, state, national, global levels) |
|  | 1. Dialogue with others (industry, policy makers, stakeholders etc.) on the nursing contribution in genomics |
|  | 1. Clear strategies are created to achieve the shared vision of nurses delivering genomic healthcare that recognises organisational need and patient/ community expectations |
| **F2 Drive within the profession** from nurse leaders to realise the vision through creating and influencing policy | 1. Nurse leaders who champion nursing roles in genomic healthcare are visible and identifiable at all levels (within and outside the clinical genomics specialty) |
|  | 1. Nurses in leadership roles (irrespective of clinical specialty) advocate for nurses delivering genomic healthcare |
|  | 1. Nurses in leadership roles work collaboratively across boundaries to generate critical mass to exert maximum influence on policy-making around genomic healthcare |
| **F3 Engagement of policy-makers** (macro to micro levels) in support for the nursing role in delivering genomic healthcare | 1. Nursing examples are sought and used to inform policy creation around genomic healthcare |
|  | 1. Non-nursing leaders in genomics actively involve nurses when developing genomics healthcare strategy and policy |
|  | 1. Legislation explicitly supports nursing roles in genomic healthcare |

**Critical Success Factor A: Education and workforce.**


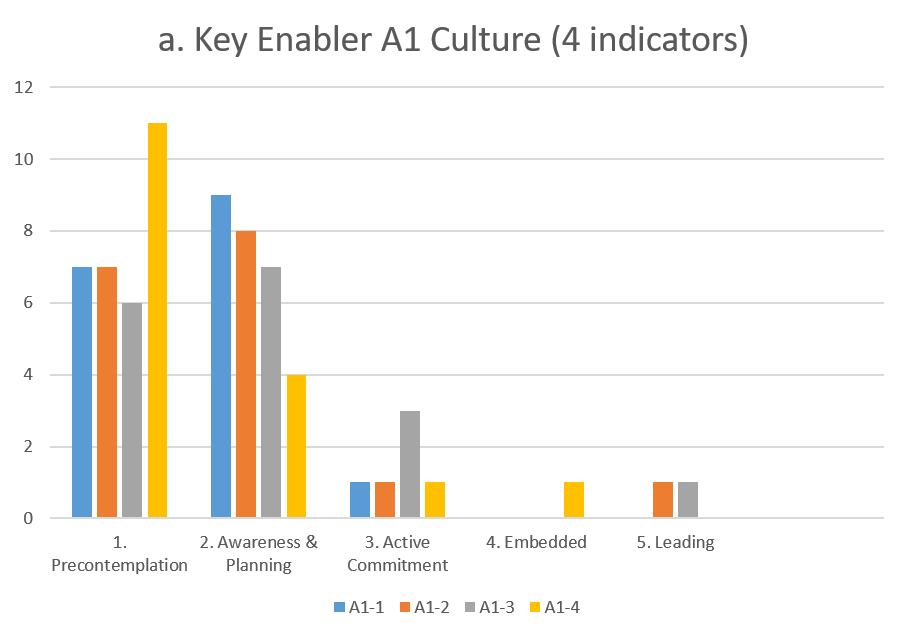


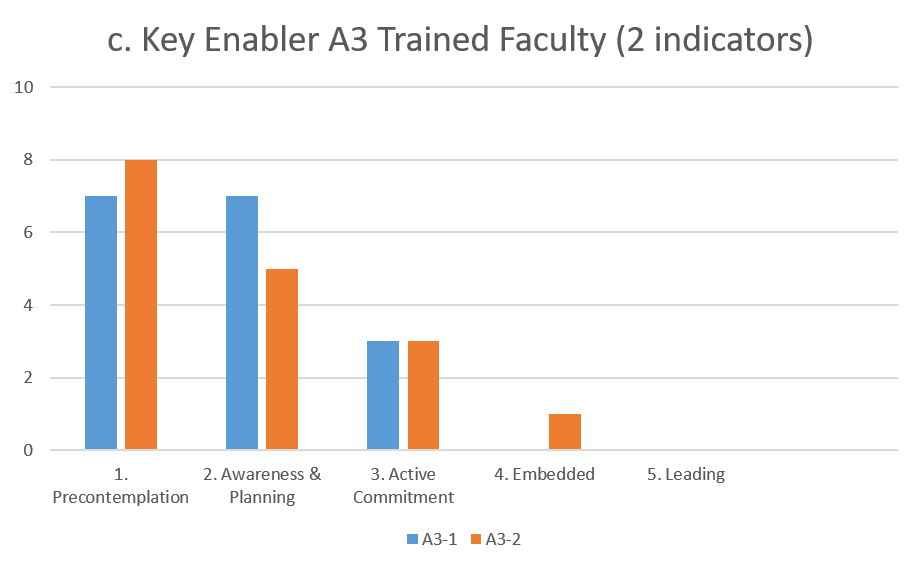


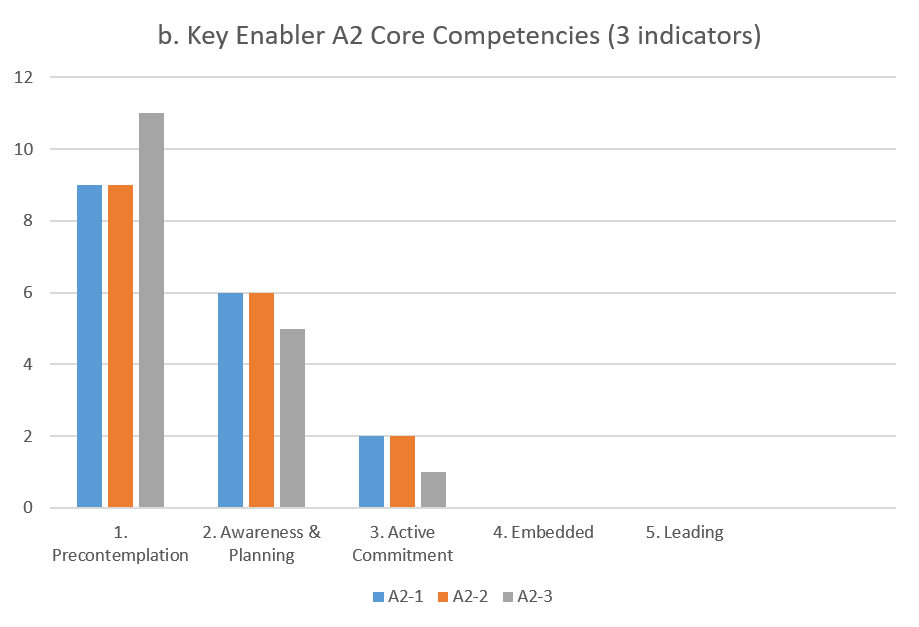


**Critical Success Factor B: Effective Nursing Practice.**


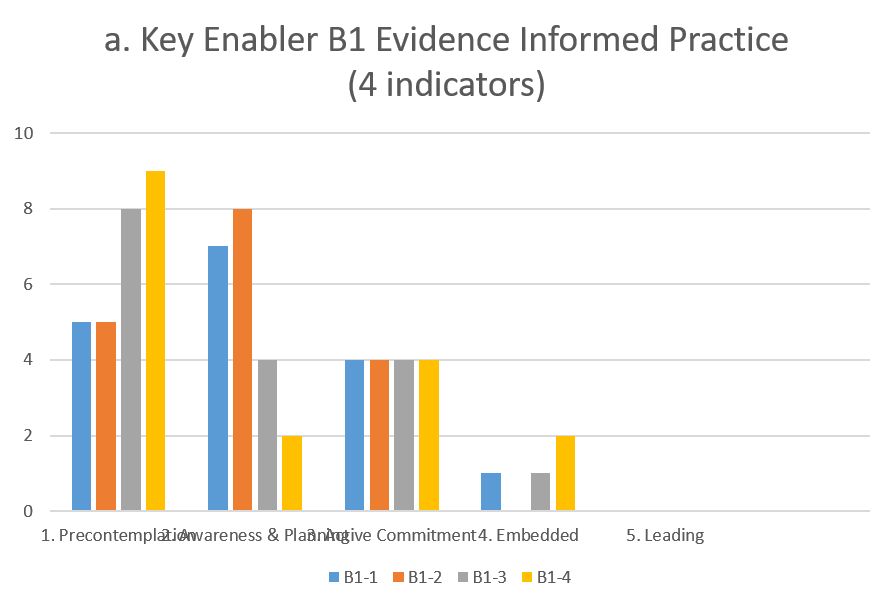


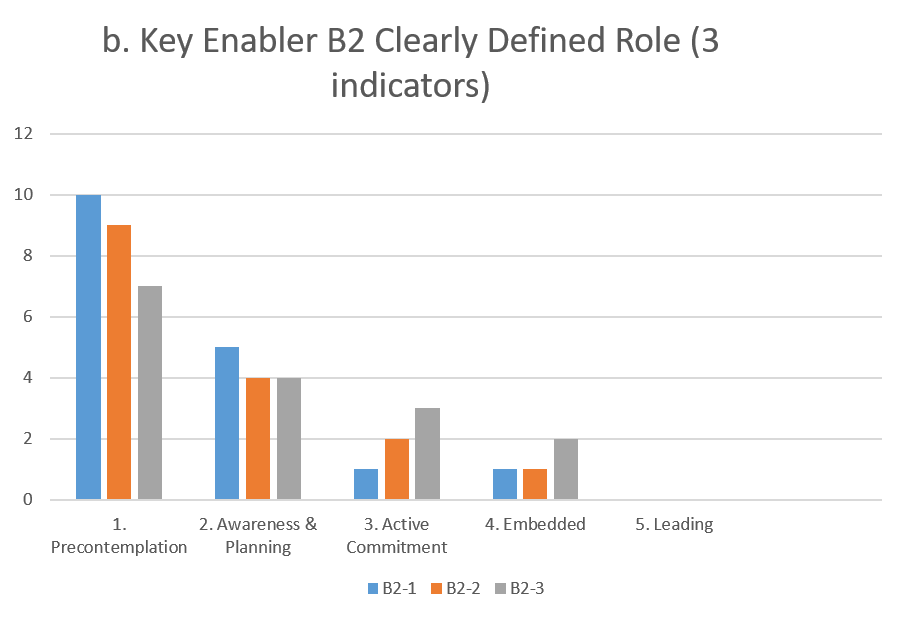


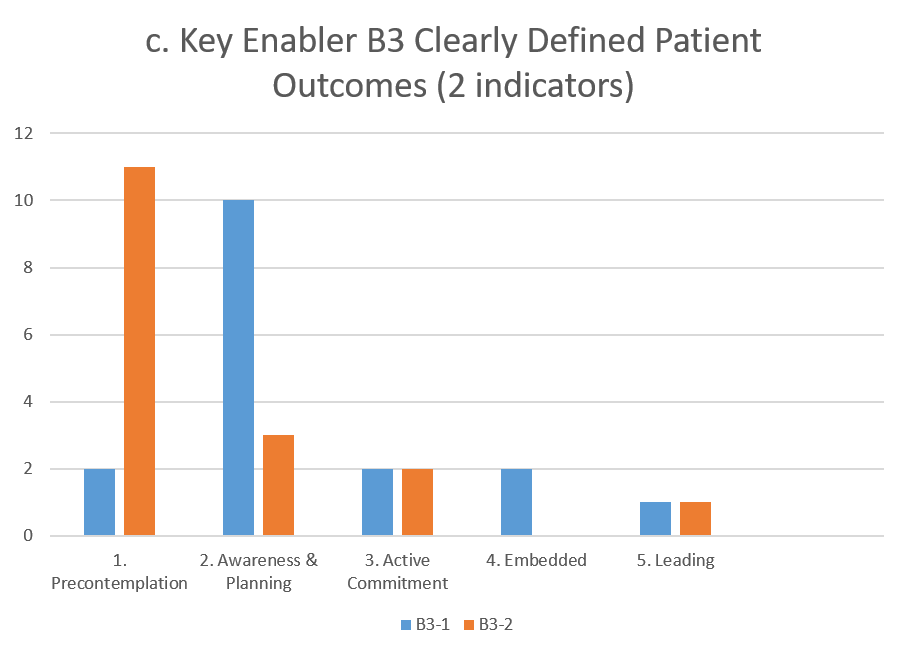


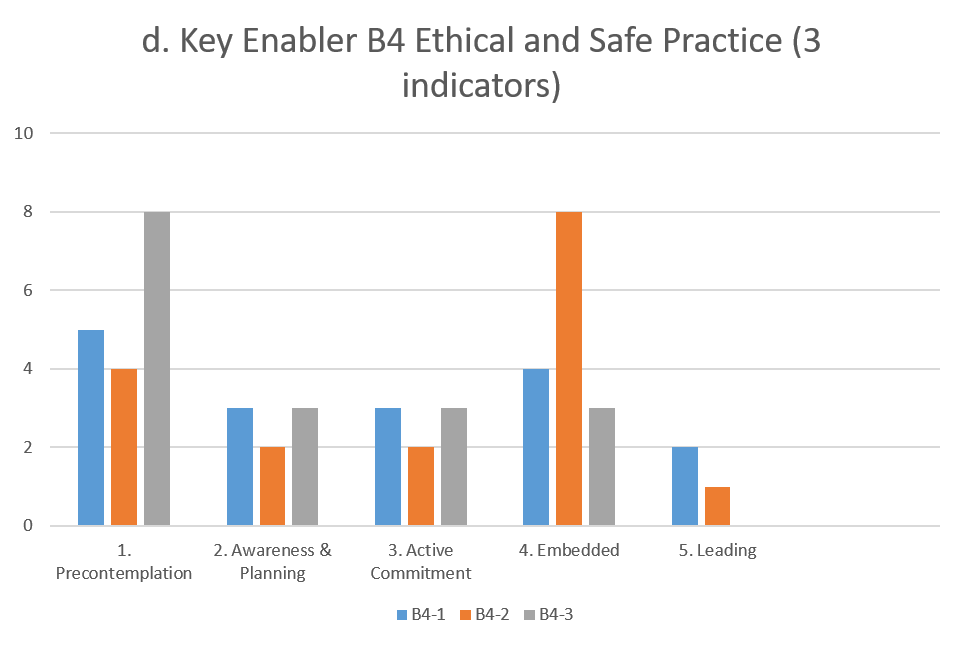


**Critical Success Factor C: Infrastructure and Resources.**


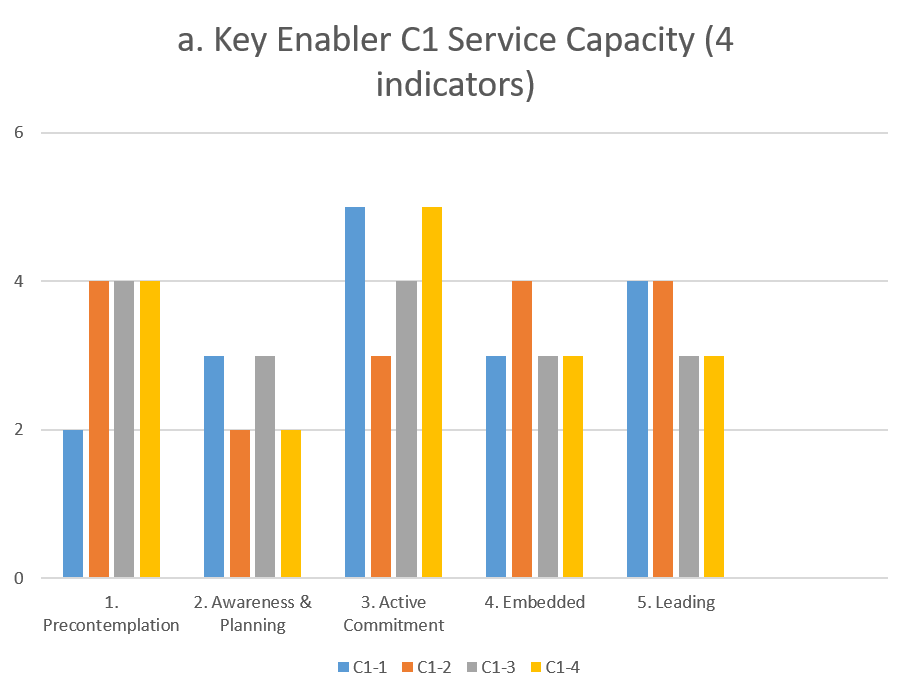


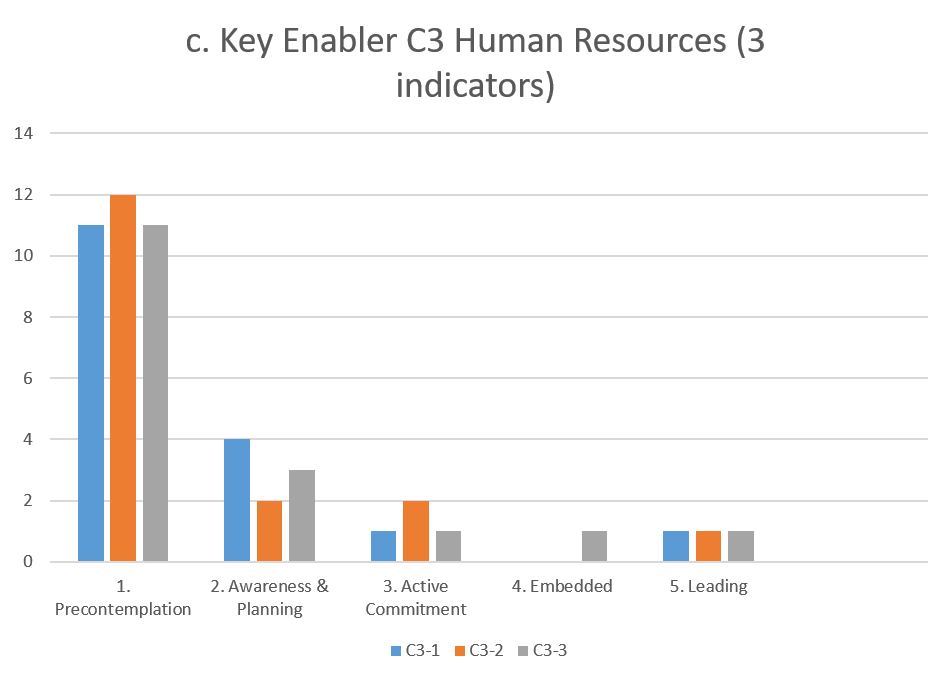


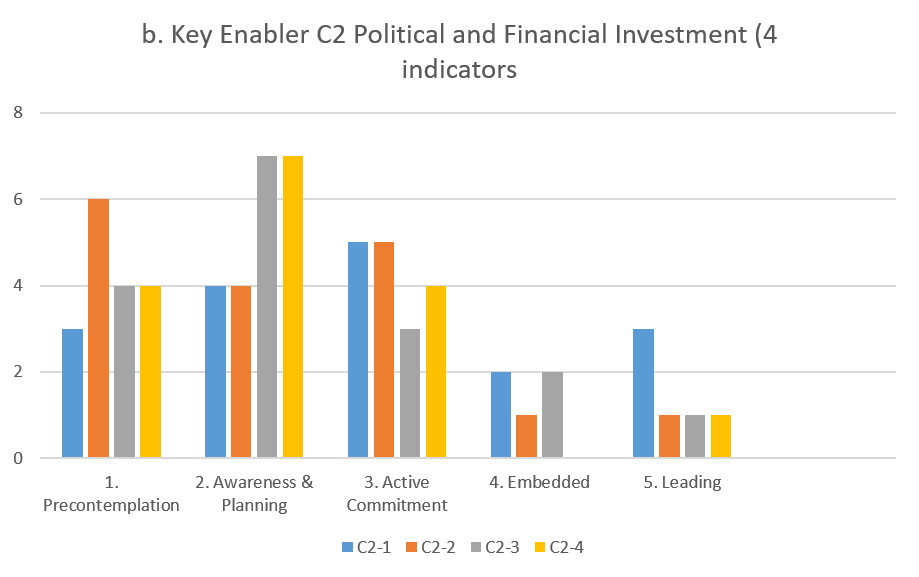


**Critical Success Factor D: Inter-professional Collaboration and Communication.**


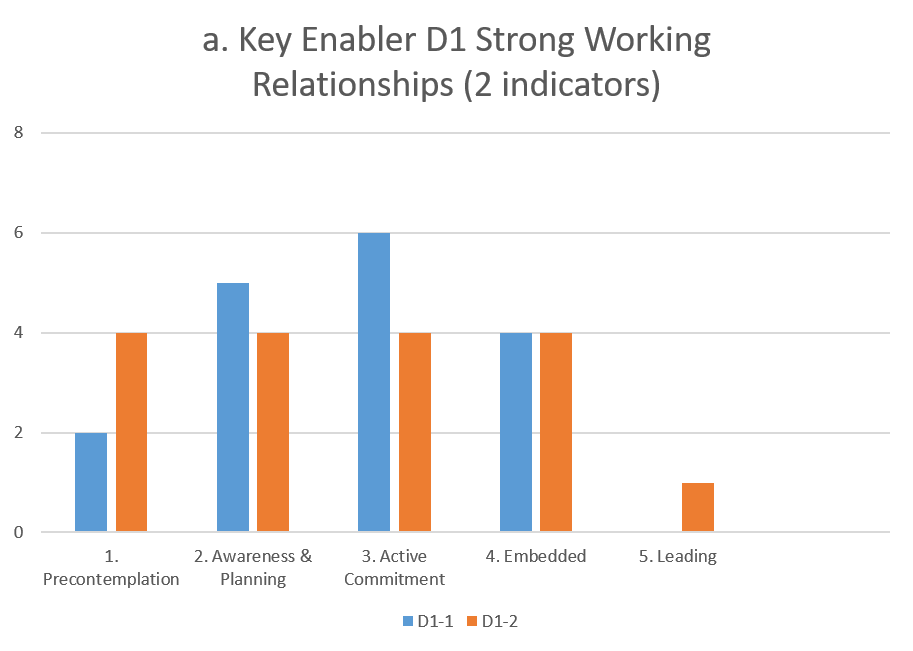


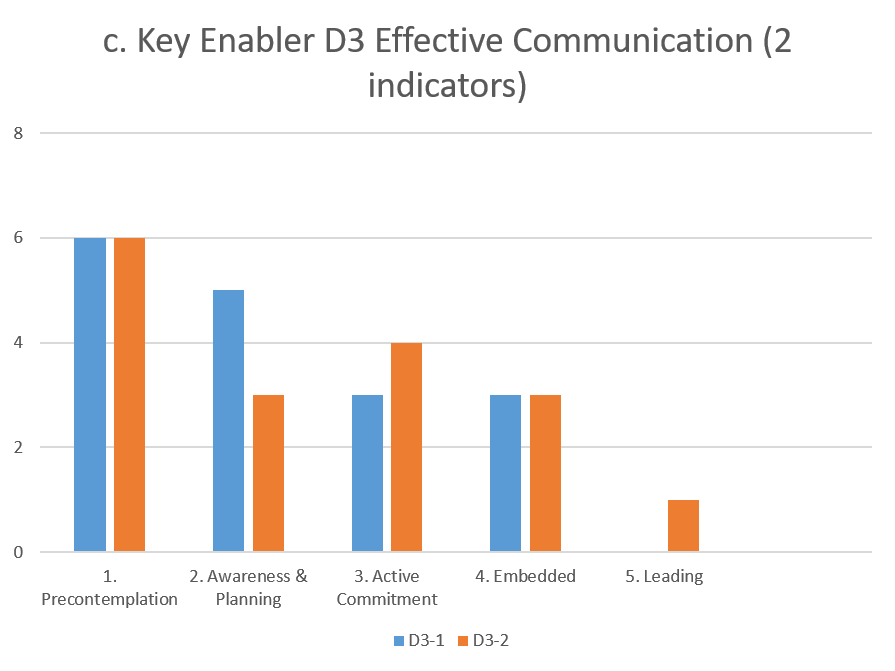


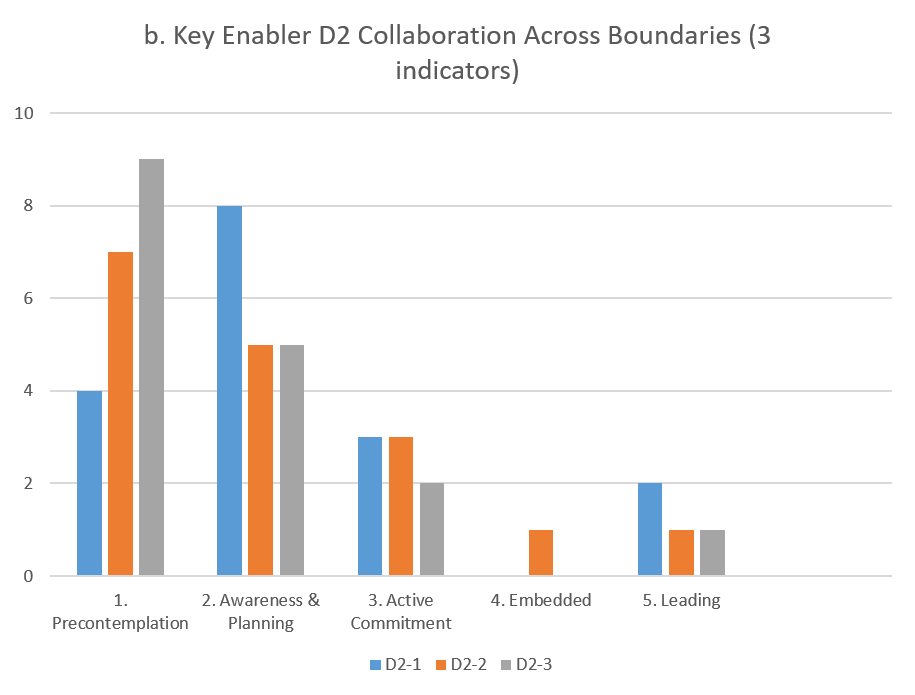


**Critical Success Factor E: Public and Patient Involvement.**


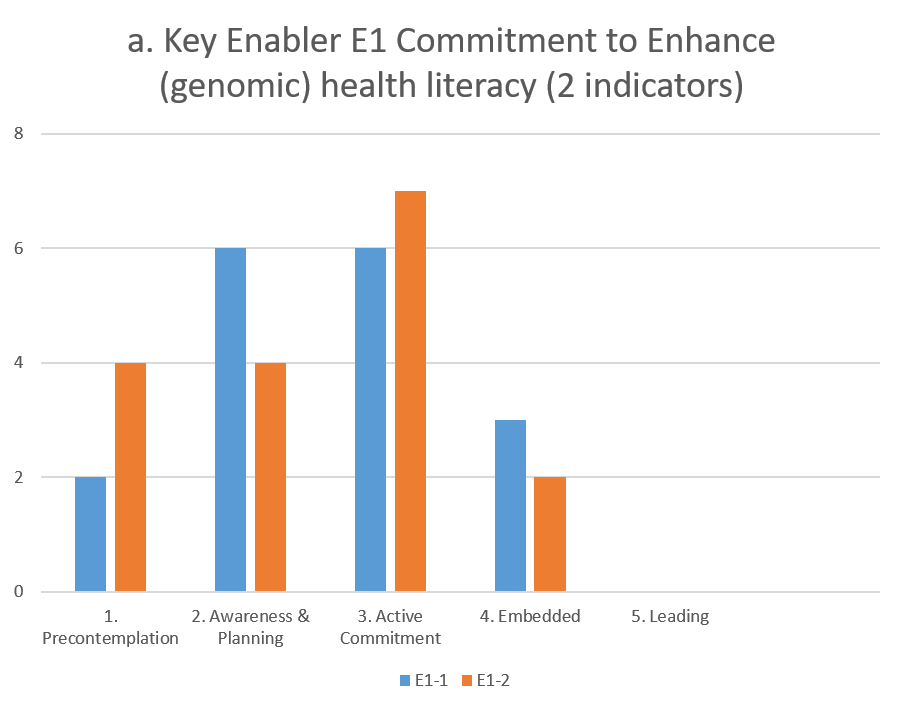


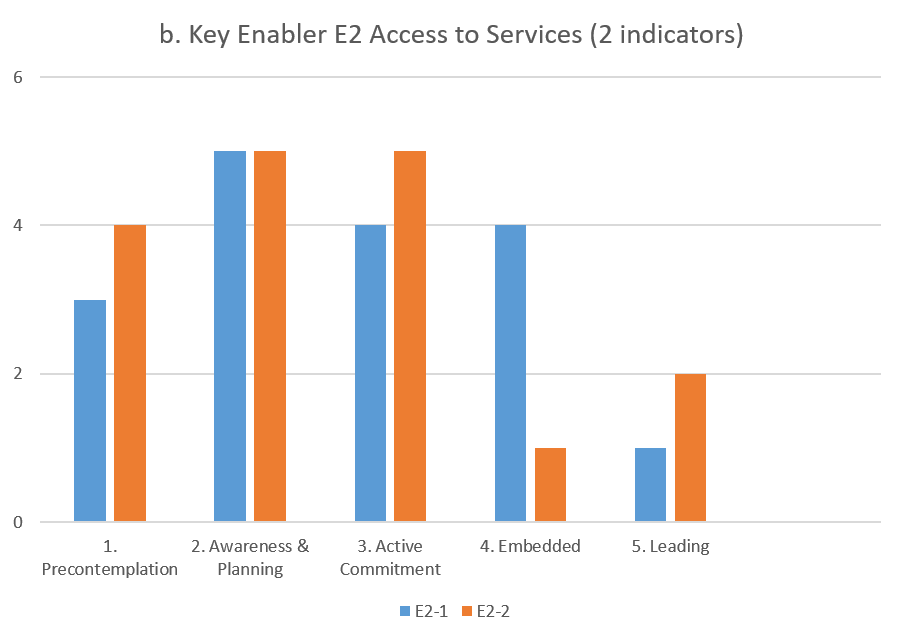


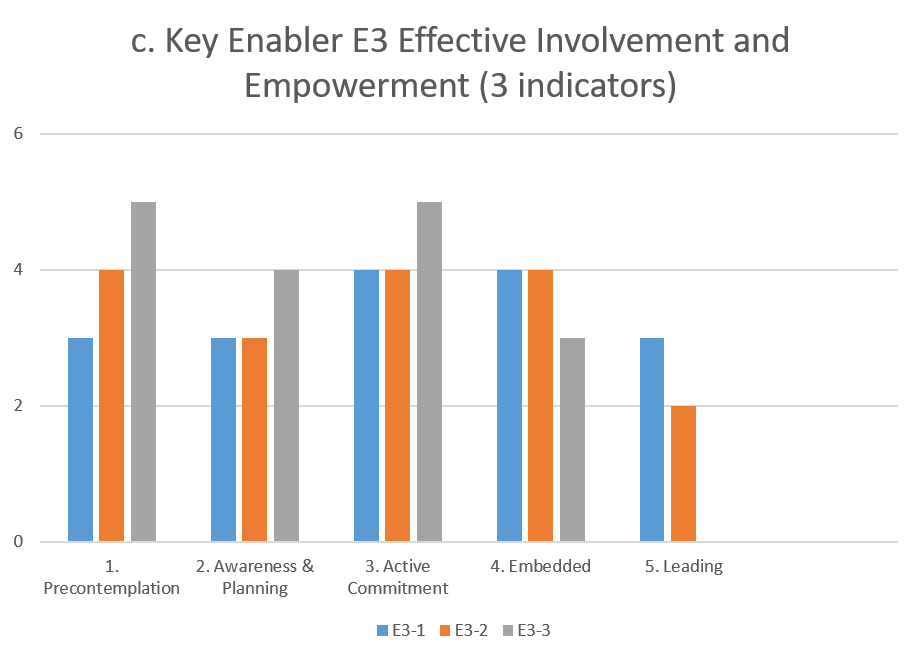


**Critical Success Factor F: Policy and Leadership.**


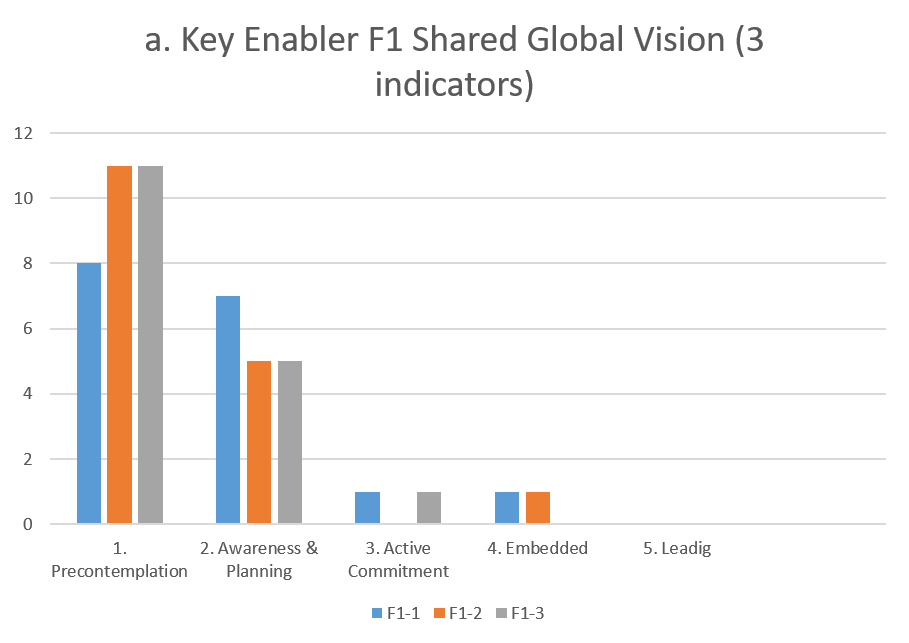


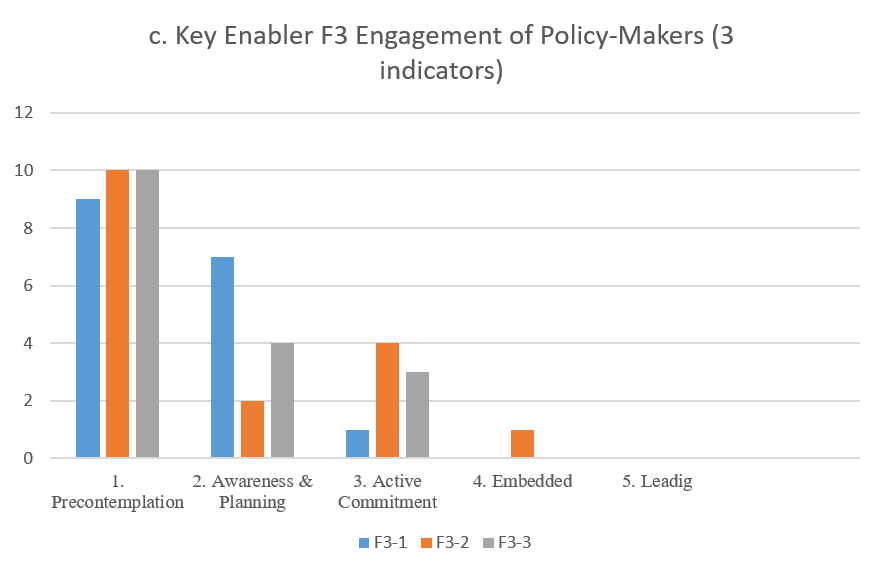


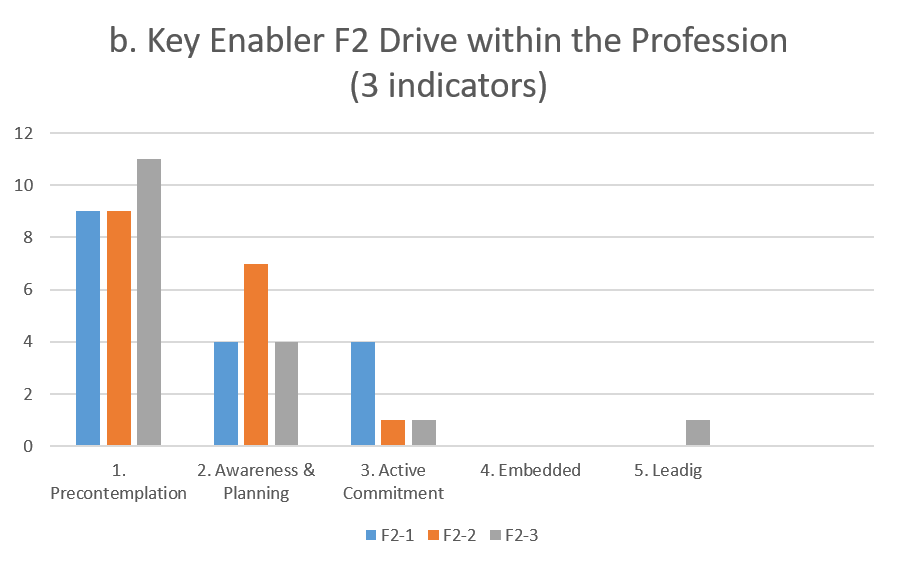


**Figure S3. Numbers of countries and spread of assessments across the indicators for each key enabler.**
